# Electron spatially fractionated radiation therapy using a clinically biocompatible 3D‐printed tungsten‐filament GRID

**DOI:** 10.1002/acm2.70567

**Published:** 2026-04-19

**Authors:** Melody Liu, Zhengzheng Xu, Hualin Zhang, Brittney Chau, Lauren Lukas

**Affiliations:** ^1^ Department of Radiation Oncology Norris Comprehensive Cancer Center University of Southern California Keck School of Medicine Los Angeles California USA

**Keywords:** electron dosimetry, GRID therapy, spatially fractionated radiation therapy

## Abstract

**Background:**

Spatially fractionated radiation therapy (SFRT) can enhance tumor control by delivering high‐dose subvolumes while sparing normal tissue and stimulating antitumor immunity. However, its application to superficial disease through electron beam radiation therapy remains limited. Electron SFRT (eSFRT) could serve as a strategy for initial tumor downsizing of ulcerated cutaneous and subcutaneous tumors by delivering a superficial, spatially heterogeneous dose, enabling subsequent conventional electron therapy to enhance local control.

**Purpose:**

To evaluate the dosimetric feasibility of eSFRT using a novel 3D‐printed tungsten‐filament GRID (TS) and compare it to lead sheet (LS) references.

**Methods:**

The TS GRID collimators were 3D‐printed from a tungsten alloy filament (91%–93% tungsten by weight, PLA‐based), with a hexagonal array of 27 apertures (1.5 cm diameter, 2.0 cm spacing, 1 mm thickness) in a 10 × 10 cm^2^ sheet. LS GRIDs (1.5 and 3.0 mm thick) were used as reference. GRIDs were placed directly on water and anthropomorphic phantoms. Dosimetric measurements for GRID field properties, including percent depth dose (PDD) and crossbeam plane dose profiles at various depths, were performed in a water tank phantom for 6, 9, and 12 MeV electron beams. Key parameters, such as depths of maximum dose (d_max_) and doses at 90% (d90) and 50% (d50), were determined. Peak‐to‐valley dose ratios (PVDRs) were also evaluated and compared with data from an anthropomorphic phantom.

**Results:**

The TS GRID collimator produced highly heterogeneous superficial dose distributions with PVDR > 2.0 at depths > 10 mm for 6–9 MeV, supporting spatially fractionated dose delivery. PVDR reductions on curved phantom surfaces were minimal (<3%). TS demonstrated reproducible geometry, mechanical stability, and biocompatibility, enabling direct placement on superficial tumors. Compared to previously reported tungsten‐based composite GRID collimators, including tungsten functional paper (TFP) and tungsten‐containing rubber (TCR), TS achieved higher PVDR at clinically relevant depths. LS GRID collimators achieved slightly higher PVDR but are not suitable for clinical use directly on skin.

**Conclusions:**

This study demonstrates the feasibility of eSFRT using a 3D‐printed TS GRID collimator to deliver superficial, spatially heterogeneous doses. TS provides reproducible geometry, mechanical stability, and biocompatibility, making it a clinically translatable alternative to lead, TFP, and TCR GRID collimators warranting further preclinical and clinical investigation.

## 1 INTRODUCTION

Ulcerated or fungating cutaneous and subcutaneous tumors, including recurrent skin cancer, cutaneous angiosarcoma, and extensive dermal metastases, are challenging to treat with conventional electron therapy due to irregular, disrupted surfaces that make uniform dose delivery difficult, leading to marginal misses and persistent tumor at the skin surface. Many of these lesions extend through the epidermis, rendering electron dose buildup ineffective for sparing normal tissue. Conventional electron therapy is limited regarding the safe delivery of high ablative doses due to excessive toxicity, particularly in previously irradiated tissue and for radioresistant histologies.^1^, ^2^, ^3^

Spatially fractioned radiation therapy (SFRT) offers a solution by delivering high‐doses to discrete subvolumes while sparing intervening and surrounding tissue^4^, ^5^ Linear accelerator (LINAC)‐based SFRT using a physical GRID collimator or lattice volumetric modulated arc therapy (VMAT) has shown improved control of bulky tumors without significantly increasing toxicity.^6^ Additionally, SFRT may stimulate immune responses, including increased tumor infiltration by immune cells and systemic activation of T cells.^7^ While photon SFRT improves bulky‐tumor control, superficial lesions will require electron‐based SFRT (eSFRT) to exploit rapid dose falloff.^8^, ^9^ Prior eSFRT collimators include metal alloy cutouts^10^ tungsten‐containing‐rubber (TCR)^9^ and tungsten functional paper (TFP).^11^ TCR and TFP are limited by low density and potentially inconsistent aperture reproducibility. This study evaluates a 3D‐printed tungsten‐filament GRID that addresses these limitations by using a high tungsten content (91%–93%) for robust PVDR, 3D printing for precise, reproducible apertures, and PLA‐based matrix for mechanical stability and clinical biocompatibility, allowing direct placement on ulcerated or irregular lesions. The primary clinical application of eSFRT is the treatment of ulcerated cutaneous/subcutaneous tumors in which (a) complete skin sparing is not desired, (b) a bolus is routinely required and therefore standard megavoltage electron beam skin sparing is already eliminated, and (c) large treatment fields produce significant acute skin toxicity with conventional uniform electron therapy. By placing the applicator (e‐GRID collimator) directly onto the target (skin lesion), setup uncertainty is reduce, thus improving dose delivery consistency. Direct skin placement of an eSFRT GRID is utilized in this study to minimize geometric penumbra and ensure precise spatial dose modulation where complete skin sparing is not desired within the high‐dose vertices. There is partial sparing of the skin and associated vasculature and immune cells within the low‐dose regions under the GRID, which could possibly stimulate biologic/bystander effects.

Effective eSFRT collimator design requires experimental validation to ensure sufficient target coverage, based on depth‐dose (PDD) profiles, and to achieve an adequate peak‐to‐valley dose ratio (PVDR) from lateral dose profiles.^12^ This study evaluates the feasibility of a novel 3D‐printed TS GRID collimator for eSFRT. Dosimetric properties were assessed using both water and anthropomorphic phantoms, and the results were compared to LS reference GRID collimators and published TCR and TFP GRID collimators.

## 2 METHODS

### 2.1 Tungsten and lead GRID collimators

The TS GRID collimator of 1 mm thickness was created by 3D printing using tungsten alloy comprised of tungsten (91%–93% by weight), a binding additive (proprietary), and polylactic acid (Rapid 3DShield Tungsten Filament^®^, Rapid 3DShield, LLC, Stoughton, WI, USA) with physical properties summarized in Table 1. The LS collimators were created using lead sheets (Radiation Products Design, Albertville, MN, USA) in thicknesses of 1.5 mm and 3.0 mm. All GRID collimators were produced by the Dornsife‐Viterbi Machine Shop at the University of Southern California.

**TABLE 1:** Physical properties of tungsten‐filament.

Item	Value
Specific gravity	7.80 g/cc
Tensile properties	•Strength: 23.3 MPa •Elongation: 7.57% •Modulus: 5,150 MPa
Flexural properties	•Strength: 45.5 MPa •Modulus: 3,550 MPa
Other properties	•Impact Strength: 95.2 J/m •Deflection Temperature: 56°C

**FIGURE 1:**
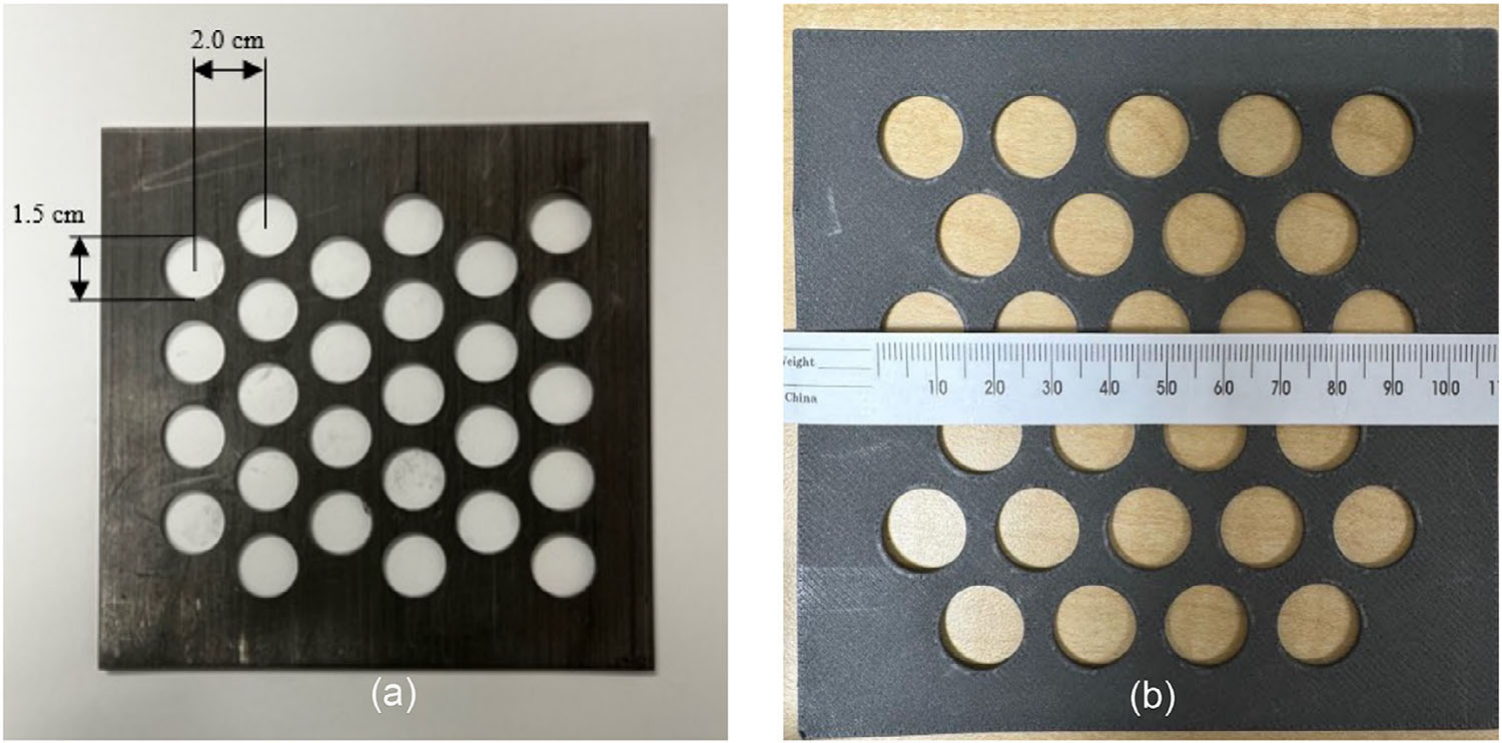
Schema of the GRID collimators. The aperture diameter was 1.5 cm and the center‐to‐center spacing of the holes were 2.0 cm for a total of 27 holes in a 10 × 10 cm^2^ sheet. (a) lead sheet (LS) GRID collimator; (b) tungsten sheet (TS) GRID collimator.

There was no post sintering of the print. The aperture diameter of the holes was 1.5 cm and the center‐to‐center spacing of the holes was 2.0 cm for a total of 27 holes in 10 × 10 cm^2^ sheets. The schema of the GRID collimators is shown in Figure 1. The selected GRID geometry (hexagonal pattern with 1.5 cm diameter apertures and 2.0 cm center‐to‐center spacing) was chosen based on prior electron and photon GRID therapy literature, experience reported in the literature^10^, ^13^ balancing achievable PVDR, peak and valley dose covered areas, mechanical stability, and clinically practical field coverage for superficial lesions. This geometry was designed to achieve PVDR values ≥ 2.0 at shallow depths relevant to cutaneous and subcutaneous tumors while maintaining sufficient peak coverage and manageable fabrication constraints using tungsten‐filament 3D printing. The dosimetric characteristics of the LS and TS collimators in eSFRT were evaluated with water tank measurements.

### 2.2 Water tank measurements

**FIGURE 2:**
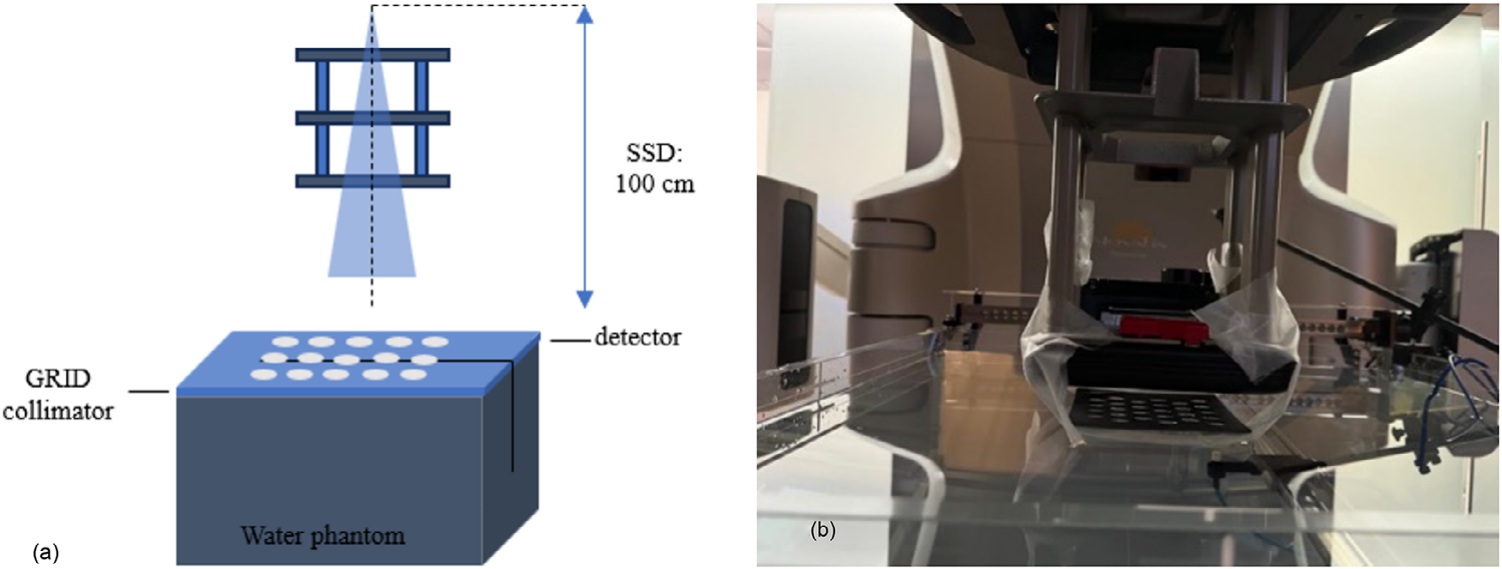
Diagram of the experimental set up for film dosimetry utilizing a water bath phantom with film placed parallel to the beam's central axis for PDD and lateral dose profile measurements. (a) Schema; (b) Experimental set up.

The characterization of the GRID field included: (1) measuring the PDD profiles; (2) measuring the depths of maximum percent depth dose (d_max_); (3) acquiring the inline and crossline dose profiles at different depths; (4) measuring the PVDR at different depths; (5) measuring the output factors (OF) at d_max_. The PDD of the central hole of the GRID collimator center and the dose profiles were measured with PTW BEAMSCAN system (Freiburg, Germany) and a microdiamond detector (PTW Freiburg, Germany). The microdiamond detector's long‐axis was aligned to the beam direction in vertical orientation where the effective volume of the detector was facing the beam entrance direction. The geometry for the measurements of the depth and lateral dose profiles with the collimators is shown in Figure 2.

Electron beams of nominal energy 6, 9, and 12 MeV on a linear accelerator (Truebeam STX, Varian Medical Systems, Palo Alto, CA, USA) with a 10 × 10 cm^2^ electron applicator and 100 cm source to surface distance (SSD) were used for the dosimetric parameter measurements of the open and GRID beams. The eSFRT was generated by LS and TS GRID collimators placed on the surface of the water phantom. The GRID was placed on the surface as mounting the GRID at the applicator exit plan (∼6 cm above surface) produces unacceptable blurring of the peak‐valley pattern due to electron scatter.

The d_max_, 90% dose (d90) and 50% dose (d50) in water were evaluated from the scanned PDD curves. Then, the lateral dose profiles were measured at d_max_ and d90. The PVDR, defined as dose ratios of the open to the blocked areas, were evaluated at various depths.^9^

### 2.3 Film based PVDR measurements using the anthropomorphic phantom

EBTXD Gafchromic films (Ashland company, Bridgewater, NJ, USA) were used, which have been shown to have a linear response curve for electron beams up to 50 Gy. During calibration the films were irradiated at 100 cm SSD (source to surface distance) with a standard 10 × 10 cm^2^ electron cone. Films were placed at the d_max_ of individual beam energies in a stack of 30 × 30 cm^2^ solid water phantom slabs (Gammex, Middleton, WI, USA), ensuring at least 10 cm of back scattering from the film location. The irradiated films were held for at least two hours for image stabilization before scanning for dose reading using the film scanner Epson Expression 11000XL (Epson America, Inc., Los Alamitos, California, USA). The irradiated films were scanned in portrait orientation and red color channel to determine the dose response curve. The film analysis software was FilmQA Pro (Version 5, Ashland company, Bridgewater, NJ, USA).

**FIGURE 3:**
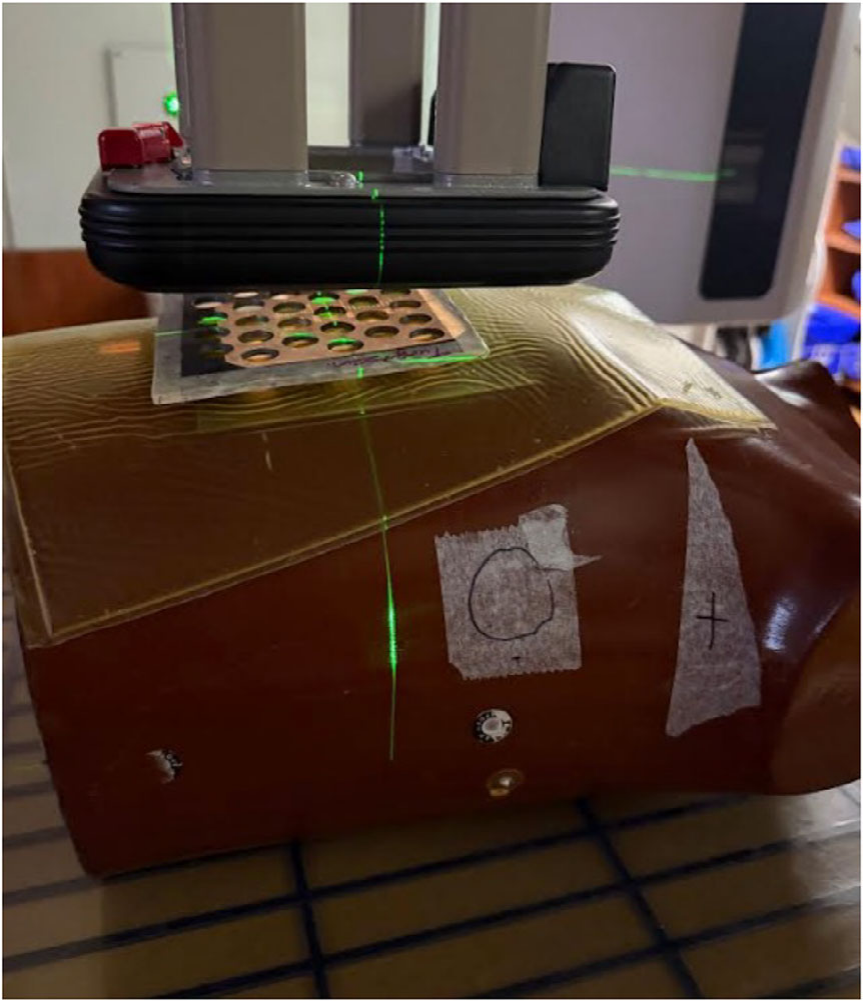
Film measurements for electron GRID sheets using the anthropomorphic phantom. The film was placed under the bolus of 1 cm thickness.

As shown in Figure 3, the anthropomorphic phantom was used to simulate a real patient's body surface curvature. The film was placed on top of the phantom and a bolus of 1 cm thickness was placed on the film and the LS or TS was placed on top of the bolus. A 10 × 10 cm^2^ applicator and SSD of 100 cm were used for electron beams of 6, 9, and 12 MeV.

### 2.4 Dose calculation

The peaks and valleys of the beam profile are dependent on the design of the collimator apertures. With the hexagonal honeycomb‐like hole pattern, the peak‐to‐peak distances are different along the inline and crossline directions, so are the valley doses. Following published studies, the PVDR is defined as

(1) $$\;PVDR_{depth}=\bar{D_{depth}^{Peak}}\;/\bar{D_{depth}^{valley}}$$

where $\bar{D_{depth}^{Peak}}$ and $\bar{D_{depth}^{valley}}$ represent the averaged peak and valley doses (across the whole beam profile) at specific depth, respectively.^15^, ^16^

When GRID collimator is used for patient care, a simple and efficient method to determine the delivered dose is through using a predetermined output factor (OF).^13^ The OF of a GRID field is defined as the output of GRID field, $M_{GRID}\left(FS,d\right)$, of specific field size (FS) at depth (d) which is normally the d_max_ of the GRID field, normalized to the output, $M_{open}(10x10,dmax$), a 10 × 10 cm^2^ cone size at d_max_ at 100 cm SSD as recommended by the TG51 guideline.^14^

(2) $$OF\;\left(FS,d\right)=\frac{M_{GRID}\left(FS,d\right)}{M_{open}\left(cone\;10x10,dmax\right)}\;$$

Then, the monitor unit (MU) needed to deliver the prescription dose at depth can be calculated by

(3) $$MU\left(FS,d\right)=\frac{Prescription\;Dose\;[cGy]}{OF\left(FS,\;d\right)**D_{o}\left(cone\;10x10,d_{max}\right)\;\left[\frac{cGy}{MU}\right]}$$

where $D_{o}\left(10x10,d_{max}\right)$ is the dose in cGy per MU of the user's beam under calibration conditions. In this study, $D_{o}\left(10x10,d_{max}\right)$ of all the energies were calibrated to 1 cGy/MU at d_max_ at 100 cm SSD. Equation 3 must be validated for the collimator at a specific machine before any clinical application.

## 3 RESULTS

### 3.1 The PDDs of the open and GRID collimated electron beams

**FIGURE 4:**
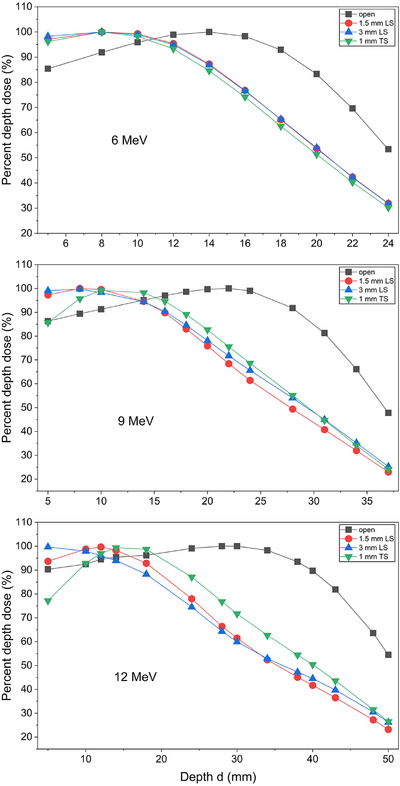
Percentage depth dose (PDD) curves for the open 10 × 10 cm^2^, 1.5 mm and 3.0 mm LS and 1 mm TS GRID collimated electron fields for 6, 9 and 12 MeV beams.

The tabulated PDDs of the open, 1.5 mm and 3.0 mm LS and 1 mm TS GRID collimated electron beams at are presented in Table 2. The PDD curves of 6, 9, and 12 MeV are shown in Figure 4.

**TABLE 2:** Percentage depth dose (PDD) data of 10 × 10 cm^2^ open and GRID electron beams with lead sheet (LS) and tungsten sheet (TS).

	Open (10 × 10 cm^2^)	1.5 mm LS GRID	3 mm LS GRID	1 mm TS GRID
**Depth(mm)**	**6 MeV**			
5	85.4	97.1	98.3	96.2
8	91.9	100.0	100.0	100.0
10	95.9	99.3	98.9	98.3
12	98.9	95.4	95.0	93.3
14	100.0	87.3	86.9	84.6
16	98.3	76.8	76.6	74.2
18	92.9	65.2	65.3	62.6
20	83.3	53.7	54.0	51.4
22	69.6	42.4	42.2	40.3
24	53.4	31.9	31.8	30.2
**Depth(mm)**	**9 MeV**			
5	86.3	97.3	99.1	85.7
8	89.4	100.0	99.7	95.7
10	91.3	99.6	98.3	99.2
14	95.1	94.6	94.4	98.2
16	97.0	89.8	90.4	94.6
18	98.6	83.0	84.6	89.1
20	99.7	75.9	78.1	82.7
22	100.0	68.4	71.7	75.5
24	99.0	61.4	65.6	68.6
28	91.8	49.4	54.0	55.1
31	81.3	40.8	45.0	44.7
34	66.1	32.0	35.2	34.2
37	47.8	23.0	25.3	24.2
**Depth(mm)**	**12 MeV**			
5	90.3	93.7	99.7	77.2
10	92.5	98.8	97.9	92.9
12	94.5	99.7	96.1	97.0
14	95.4	98.3	94.0	99.4
18	96.2	92.9	88.3	98.7
24	99.1	78.0	74.5	87.1
28	100.0	66.4	64.3	76.8
30	100.0	61.5	59.9	71.7
34	98.3	52.4	52.9	62.7
38	93.5	45.1	47.2	54.4
40	89.7	41.7	44.5	50.4
43	81.9	36.5	39.7	43.6
48	63.6	27.2	30.5	31.5
50	54.5	23.2	26.2	26.5

Measured values of depth of d_max_, d90 and d80 from the PDDs for the GRID collimated electron beams with lead and TS GRID collimators are presented in Table 3 as these values are clinically relevant for the prescribing radiation oncologist. It was found that the 1.5 mm LS demonstrated the most significant changes in PDDs when the energy increased from 9 MeV to 12 MeV and d_max_ shifted to a deeper depth. However, for 3 mm LS, d_max_ shifted to a shallower depth when energy increased.

**TABLE 3:** Measured d_max_, d90, and d50 from the PDDs for the GRID collimated 6 MeV, 9 MeV, and 12 MeV electron beams with lead sheet (LS) and tungsten sheet (TS).

		Depth (mm)		
Energy (MeV)	Collimator	d_max_	d90	d50
6 MeV	LS 1.5 mm	8.2	13.3	20.6
	LS 3.0 mm	8.0	13.2	20.7
	TS 1.0 mm	8.0	12.7	20.2
	Open (10 × 10 cm^2^)	13.0	17.4	23.6
9 MeV	LS 1.5 mm	8.4	15.9	27.7
	LS 3.0 mm	8.1	16.2	29.3
	TS 1.0 mm	11.6	17.7	29.5
	Open (10 × 10 cm^2^)	20.8	27.4	35.7
12 MeV	LS 1.5 mm	10.6	19.5	35.3
	LS 3.0 mm	7.8	17.0	36.0
	TS 1.0 mm	15.6	22.9	40.1
	Open (10 × 10 cm^2^)	28.5	39.3	50.3

### 3.2 Beam profiles of GRID beams and PVDR

**FIGURE 5:**
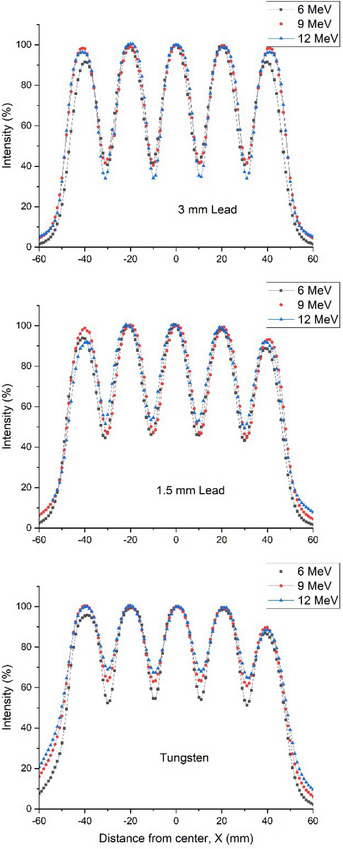
GRID electron beam 2D dose profiles (at d90) using 1.5 mm lead GRID sheet, 3.0 mm thickness lead GRID sheet, and tungsten GRID sheet.

2D beam profiles of different energies for LS and TS collimators at d_max_ and d90 were shown in Figure 5.

**FIGURE 6:**
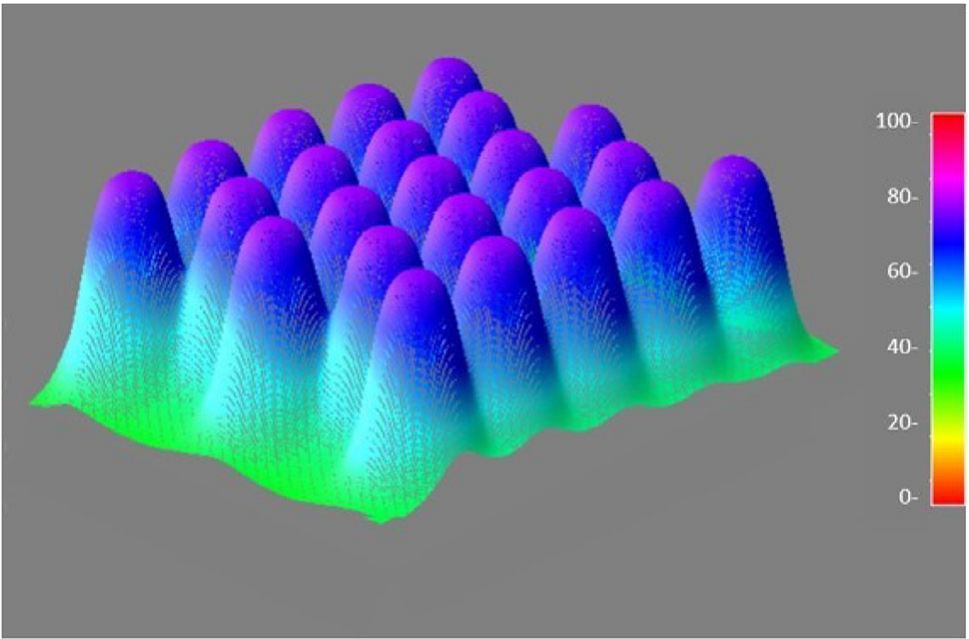
A representative 3D dose profile from a film measurement for a 3 mm lead GRID using 6 MeV at the depth of d_max_.

Figure 6 is a representative 3D dose profile which is from a film measurement for a 3 mm lead GRID using 6 MeV at the depth of d_max_. A clear peak and valley dose distribution was seen. Other collimators gave similar results.

The profiles demonstrated pronounced differences in Tungsten collimators. However, for lead applicators, when the energy was increased from 6 to 12 MeV, the valley dose exhibited less significant variation, provided the dose was normalized at the d90 of the central hole. The output factors of the electron fields at d90 and d_max_ for LS and TS GRID collimators were presented in Table 4.

**TABLE 4:** Output factors of 10 × 10 cm^2^ electron GRID fields at depth for lead sheet (LS) and tungsten sheet (TS).

	Output factors								
	6 MeV			9 MeV			12 MeV		
Depth (mm)	LS ‐ 1.5 mm	LS ‐ 3.0 mm	TS ‐ 1.0 mm	LS ‐ 1.5 mm	LS ‐ 3.0 mm	TS ‐ 1.0 mm	LS ‐ 1.5 mm	LS ‐ 3.0 mm	TS ‐ 1.0 mm
5	0.928	0.945	0.945	0.944	0.985	0.857	0.925	0.992	0.770
10	0.951	0.951	0.966	0.971	0.976	0.990	0.986	0.975	0.929
15	0.785	0.801	0.780	0.902	0.911	0.961	0.965	0.916	0.988
20	0.494	0.509	0.506	0.749	0.768	0.830	0.853	0.846	0.956
d_max_	0.961	0.964	0.979	0.974	0.988	0.992	0.996	0.993	0.998
	(*d* = 8.2 mm)	(*d* = 8.0 mm)	(*d* = 8.0 mm)	(*d* = 8.4 mm)	(*d* = 8.1 mm)	(*d* = 11.6 mm)	(*d* = 10.6 mm)	(*d* = 7.8 mm)	(*d* = 15.6 mm)
d90	0.869	0.871	0.883	0.881	0.891	0.898	0.901	0.895	0.922
	(*d* = 13.3 mm)	(*d* = 13.2 mm)	(*d* = 12.7 mm)	(*d* = 15.9 mm)	(*d* = 16.2 mm)	(*d* = 17.7 mm)	(*d* = 19.5 mm)	(*d* = 17.0 mm)	(*d* = 22.9 mm)

The mean PVDRs at depths ranging from surface to 20 mm were presented in Table 5. The PVDRs of LS exceeded 2.0 for all three energies at depths less than 15 mm. For TS, PVDRs were greater than 2.0 when using lower energies (6 and 9 MeV) at depths below 10 mm. However, for 12 MeV, the PVDR remained below 2.0 at all depths.

**TABLE 5:** PVDRs of electron GRID fields using lead sheets (LS) of 1.5 and 3 mm thicknesses and tungsten sheet (TS) of 1 mm thickness at various depths.

	PVDR								
	6 MeV			9 MeV			12 MeV		
Depth (mm)	LS ‐ 1.5 mm	LS ‐ 3.0 mm	TS ‐ 1.0 mm	LS ‐ 1.5 mm	LS ‐ 3.0 mm	TS ‐ 1.0 mm	LS ‐ 1.5 mm	LS ‐ 3.0 mm	TS ‐ 1.0 mm
0	2.81	6.28	1.41	1.88	5.91	1.25	1.26	2.88	1.21
5	4.07	4.56	2.49	3.03	7.07	1.92	2.18	4.23	1.70
10	3.11	5.02	2.38	2.86	4.93	2.14	2.48	4.75	1.91
15	2.05	2.11	1.97	2.25	2.72	1.71	2.22	3.45	1.93
20	1.25	1.41	1.10	1.54	1.59	1.23	1.98	2.29	1.70
d_max_	4.31	5.88	2.56	3.17	6.96	2.04	2.51	5.08	1.92
	(*d* = 8.2 mm)	(*d* = 8.0 mm)	(*d* = 8.0 mm)	(*d* = 8.4 mm)	(*d* = 8.1 mm)	(*d* = 11.6 mm)	(*d* = 10.6 mm)	(*d* = 7.8 mm)	(*d* = 15.6 mm)
d90	2.31	2.50	1.95	2.22	2.47	1.65	2.01	2.94	1.55
	(*d* = 13.3 mm)	(*d* = 13.2 mm)	(*d* = 12.7 mm)	(*d* = 15.9 mm)	(*d* = 16.2 mm)	(*d* = 17.7 mm)	(*d* = 19.5 mm)	(*d* = 17.0 mm)	(*d* = 22.9 mm)

### 3.3 PVDRs measured on the anthropomorphic phantom

PVDRs of electron GRID fields measured in an anthropomorphic phantom were compared with those obtained in a water phantom (Table 6). The PVDR measured with the anthropomorphic phantom were slightly lower than those acquired in the water phantom due to the curved surface. The difference in PVDR between the two phantoms was within 3%.

**TABLE 6:** PVDRs of GRID electron fields measured on the anthropomorphic phantom.

Energy	Bolus 1 cm			Water tank measurements at 1 cm depth		
(MeV)	LS ‐ 1.5 mm	LS ‐ 3.0 mm	TS ‐ 1.0 mm	LS ‐ 1.5 mm	LS ‐ 3.0 mm	TS ‐ 1.0 mm
6	3.06	4.98	2.31	3.11	5.02	2.38
9	2.78	5.03	2.08	2.86	4.93	2.14
12	2.42	4.72	1.86	2.48	4.75	1.91

## 4 DISCUSSION

This study demonstrates that a 3D‐printed TS GRID collimator is feasible for eSFRT and could be further evaluated for use in initially downsizing ulcerated cutaneous and subcutaneous tumors, where conventional electron therapy is limited. eSFRT has potential for treating advanced, bulky, and recurrent skin cancers.^15^ Conventional radiation is limited by toxicity, often causing skin inflammation and damage mediated by T‐cell driven immune responses.^16^, ^17^, ^18^, ^19^, ^20^, ^21^ It is postulated that, eSFRT may reduce toxicity by sparing normal vasculature and immune cells in the low‐dose regions under the collimator, allowing for effective tumor debulking and subsequent conventional radiation. Additionally, spatial dose escalation may enhance antigen presentation and immune activation.^7^ Combining eSFRT with immunotherapy, such as immune checkpoint inhibitors or cytokine therapy, could potentially further amplify antitumor effects and overcome immune evasion.^7^ As such, eSFRT is not being proposed as a replacement for conventional electron beam therapy, but as a niche tool for complex superficial presentations poorly served by existing modalities.

The key findings are that the TS GRID achieved a PVDR ≥ 2.0, supporting spatially fractionated dose delivery using 6–12 MeV electrons relevant for superficial oncology indications, with minimal PVDR reduction on a curved surface (<3%) which indicates applicability to irregular surfaces. The use of an anthropomorphic phantom to evaluate spatial modulation of electrons under realistic curvature has not been previously reported. Both LS and TS achieved PVDRs ≥ 2.0 at d_max_, though TS fell below 2.0 at d90. In photon‐based SFRT, PVDR > 2.0 is generally considered sufficient for immune modulation and tumor control^22^, ^23^, ^24^ although lower ratios (>1.5) may be acceptable when integrated into a stereotactic body radiotherapy (SBRT) plan.^25^ Prior studies reported lower PVDRs for TP (1.32 at d_max_, 1.20 at d90)^11^ and TCR (1.77 at d_max_, 1.51 at d90).^9^ In contrast, our TS and LS collimators demonstrated higher superficial PDDs and improved PVDRs, suitable for treating cutaneous tumors. The TS GRID pattern and electron energies evaluated in this study (6–12 MeV) are intended for superficial cutaneous and subcutaneous tumors extending from the surface to approximately 10–20 mm in depth. Based on the measured PDDs and PVDRs, 6 and 9 MeV electrons achieved PVDR > 2.0 cm at depths up to ∼10 mm, making them well suited for superficial and ulcerated lesions. Specifically, 6 MeV electrons achieved PVDR ≥ 2.0 at depths up to approximately 10 mm, with a maximum PVDR of 2.56 at dmax (8.0 mm) while 9 MeV electrons achieved PVDR values approaching or exceeding 2.0 up to 10–12 mm, with a PVDR of 2.04 at d_max_ (11.6 mm). The 12 MeV beam provided deeper penetration but reduced PVDR, although still in line with ratios > 1.5 that have been considered acceptable in photon plans.^25^ TS offers reproducible apertures, mechanical stability, and biocompatibility, allowing safe direct placement. To maximize patient safety, further medical‐grade encapsulation with silicone could be considered to prevent direct contact with skin. The mass of the TS GRID collimator (< 350 g) poses no mechanical safety risk.

Limitations of the study include evaluation of only one GRID geometry and aperture size using a 10 × 10 cm^2^ sheet. This study represents an initial feasibility and proof‐of‐concept evaluation rather than a full geometric optimization and thus we limited measurements to the 10 × 10 cm^2^ applicator, however, larger applicators (20–25 cm) and off‐normal incidence setups to further mimic curved anatomy need to be further evaluated. The 10 × 10 cm^2^ GRID sheet with 27 apertures is suitable for lesions several centimeters in lateral extent, such as plaque‐like or fungating tumors; larger tumors may be addressed using sequential placement or larger GRID applicators. GRID design parameters, including aperture size, pattern, spacing, thickness, and material type, can be further optimized by clinical trials for specific clinical indicators and beam energies. Optimization studies using Monte Carlo simulations and experimental validation are considered helpful. Further research is also needed to explore the contributions of various geometry dimensions to biological mechanisms of eSFRT, including bystander effects and cytokine signaling.^26^

The GRID collimators were intentionally placed directly on the water phantom surface to preserve spatial dose modulation and minimize lateral electron scatter. When electron GRID collimators are mounted at the applicator's exit plane (∼6 cm above the surface), significant peak‐valley blurring could occur due to air scatter and electron divergence, particularly for low‐energy beams such as 6 MeV. Direct surface placement minimizes geometric penumbra, maintains peak‐valley contrast, and more accurately implements the intended clinical use for ulcerated and often irregular‐shaped superficial tumors, where skin sparing is not desired and bolus is routinely applied to increase the skin dose.

Regarding reproducibility, direct surface placement improves setup consistently by eliminating air gaps and positional uncertainty associated with applicator‐mounted devices. The TS GRID demonstrated mechanical rigidity, dimensional stability, and reproducible aperture geometry, enabling reliable and repeatable positioning. Repeated measurements showed consistent PDDs, profiles, and PVDR values within the measurement uncertainty, including for 6 MeV electrons, which are most sensitive to scatter effects. Regarding potential photon contamination, since the GRID thicknesses are only 1–3 mm, the expected bremsstrahlung contribution is very small relative to the photon tail of an unmodified electron beam. Our film measurements showed no detectable increase in photon contamination beyond typical out‐of‐field electron bremsstrahlung. the majority of bremsstrahlung x‐ray was absorbed by the GRID collimator.

## 5 CONCLUSIONS

This study demonstrates that a 3D‐printed TS GRID collimator is dosimetrically feasible for eSFRT in ulcerated cutaneous and subcutaneous tumors, where conventional electron therapy is limited to deliver doses sufficient for maximal local control. TS GRID offers a high PVDR and heterogeneous superficial dosing with mechanical stability and biocompatibility and offers a clinically translatable alternative to lead, TFP, and TCR GRID collimators. TS GRID eSFRT may serve as an initial tumor debulking strategy, minimizing toxicity while enabling subsequent conventional electron therapy to maximize local control. These findings support further preclinical and clinical studies investigating the therapeutic and immunologic impact of TS GRID eSFRT.

## AUTHOR CONTRIBUTION

All authors contributed to the study concept, design, and manuscript draft revisions.

## CONFLICT OF INTEREST STATEMENT

The authors declare no conflicts of interest related to this work.

## ETHICAL STATEMENT

This study did not involve human participants, identifiable patient data, or animal subjects. All dosimetric analyses were conducted using phantom measurements that do not contain personal health information. As such, institutional review board (IRB) approval was not required. The research was performed in accordance with ethical standards for scientific integrity and data handling as outlined by the Journal of Applied Clinical Medical Physics.
